# Runaway Train: A Leaky Radiosensitive SCID with Skin Lesions and Multiple Lymphomas

**DOI:** 10.1155/2018/2053716

**Published:** 2018-05-14

**Authors:** Børre Fevang, Unn Merete Fagerli, Hanne Sorte, Harald Aarset, Håkon Hov, Marit Langmyr, Thomas Morten Keil, Ellen Bjørge, Pål Aukrust, Asbjørg Stray-Pedersen, Tobias Gedde-Dahl

**Affiliations:** ^1^K.G. Jebsen Center for Cancer Immunotherapy and K.G. Jebsen Inflammation Research Centre, Institute of Clinical Medicine, University of Oslo, Oslo, Norway; ^2^Research Institute of Internal Medicine, Oslo University Hospital, Rikshospitalet, Oslo, Norway; ^3^Section of Clinical Immunology and Infectious Diseases, Oslo University Hospital, Rikshospitalet, Oslo, Norway; ^4^Department of Oncology, St. Olav's Hospital, Trondheim, Norway; ^5^Department of Cancer Research and Molecular Medicine, Faculty of Medicine and Health Sciences, NTNU, Trondheim, Norway; ^6^Department of Medical Genetics, Oslo University Hospital, Oslo, Norway; ^7^Department of Pathology, St. Olav's Hospital, Trondheim, Norway; ^8^Department for Laboratory Medicine, Children's and Women's Health, Faculty of Medicine and Health Sciences, NTNU, Trondheim, Norway; ^9^Department of Radiology, St. Olav's Hospital, Trondheim, Norway; ^10^Department of Nuclear Medicine, St. Olav's Hospital, Trondheim, Norway; ^11^Department of Dermatology, St. Olav's Hospital, Oslo, Norway; ^12^Department of Hematology, Oslo University Hospital, Oslo, Norway; ^13^Institute for Clinical Medicine, Medical Faculty, University of Oslo, Oslo, Norway

## Abstract

The nuclease Artemis is essential for the development of T-cell and B-cell receptors and repair of DNA double-strand breaks, and a loss of expression or function will lead to a radiosensitive severe combined immunodeficiency with no functional T-cells or B-cells (T-B-SCID). Hypomorphic mutations in the* Artemis* gene can lead to a functional, but reduced, T-cell and B-cell repertoire with a more indolent clinical course called “leaky” SCID. Here, we present the case of a young man who had increasingly aggressive lymphoproliferative skin lesions from 2 years of age which developed into multiple EBV+ B-cell lymphomas, where a hypomorphic mutation in the* Artemis* gene was found in a diagnostic race against time using whole exome sequencing. The patient was given a haploidentical stem cell transplant while in remission for his lymphomas and although the initial course was successful, he succumbed to a serious* Pneumocystis jirovecii* pneumonia 5 months after the transplant. The case underscores the importance of next-generation sequencing in the diagnosis of patients with suspected severe immunodeficiency.

## 1. Introduction

The intricate enzymatic machinery needed for the recombination of T-cell and B-cell receptors is the basis of adaptive immunity and major mutations in associated genes like* RAG* and* Artemis* invariably lead to severe combined immunodeficiency with no or very limited expression of T-cells and B-cells (T-B-SCID) [1, 2]. However, hypomorphic mutations in these genes can lead to a spectrum of less severe phenotypes with a functional but reduced T-cell and B-cell repertoire, including the so-called leaky SCID [3–8]. These patients have SCID-like features but do not fill the criteria for SCID and have, at least initially, a milder and more indolent clinical course.

The Artemis protein (also known as DNA cross-link repair enzyme 1C, DCLRE1C) was first characterized as a part of the VDJ-recombination process in 2001 in a study of patients with radiosensitive SCID [2]. Importantly, the Artemis protein, as opposed to RAG, was found to be essential also for the repair of DNA double-strand breaks explaining the radiosensitive nature of the immunodeficiency. It is expressed in a wide variety of tissues, including fibroblasts, facilitating the use of fibroblast cultures in diagnostic radiosensitivity assays. Typically, complete Artemis deficiency presents itself as T-B-SCID in early infancy with life-threatening infections, diarrhea, and failure to thrive. The clinical presentation is similar to other forms of T-B-SCID, and while increased radiosensitivity heightens the suspicion of Artemis disease, genetic tests will be needed to distinguish it from, for example, DNA Ligase IV deficiency. Artemis deficiency has a recessive pattern of inheritance and has been found with increased frequency in certain population groups, including native Americans. Hematopoietic stem cell transplantation (HSCT) is the only available curative treatment, but conditioning is complicated by increased sensitivity to both radiation and alkylating agents. Radiosensitive SCID, including Artemis deficiency, is thus an obvious candidate for gene therapy [9].

The use of advanced genetic methods in diagnosis of primary immunodeficiencies has shown us that the spectrum of diseases associated with SCID genes is both wide and variable. While homozygotic null-mutations in* Artemis* have a defined phenotype, multiple publications over the last years have shown that other genetic variants in* Artemis*, including heterozygotic and compound heterozygotic mutations, can cause a range of clinical presentations. “Leaky” SCIDs are defined by low levels of T-cells with an impaired functional response and have been associated with hypomorphic mutations in SCID-associated genes like* Artemis*, which cause dysfunction but not absence of the related protein [7]. The clinical consequence of these nonclassical SCID-associated mutations is unpredictable, but any dysfunction related to DNA-repair mechanisms like Artemis is likely to have a serious and progressive course.

## 2. Case Presentation

The patient was a 23-year-old male of Turkish/Kurdish descent of consanguineous parents (second-cousins) who had 4 healthy siblings and one sister with a rare lung disease not immediately associated with the patient's condition. His parents had lost a child at a few months of age with no certain diagnosis.

**Figure 1:**
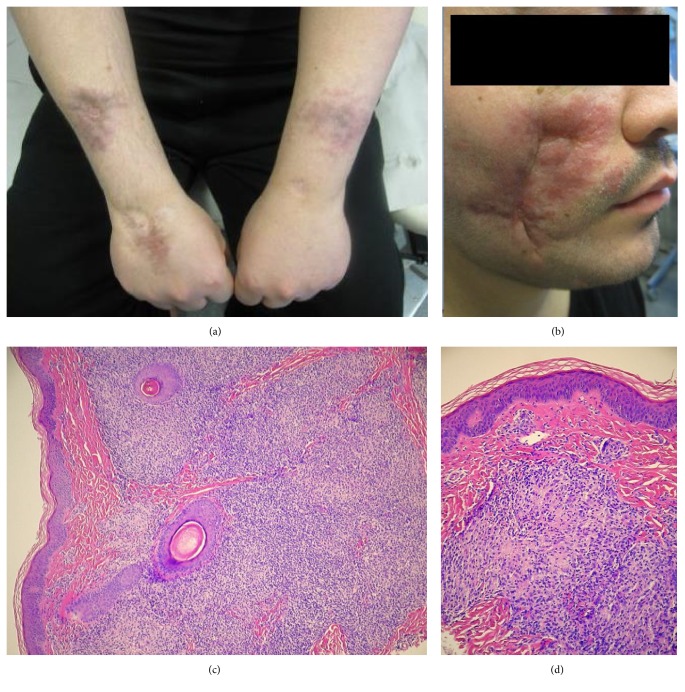
Skin lesions. (a) multiple scar-like bluish-red lesions on both arms. (b) indurated, erythematous scar-like lesion on the left cheek. (c, d) Skin biopsy showing massive lymphohistiocytic infiltration focally with formation of granulomas (H&E, 100x and 400x, resp.). There was a dominance of CD8+ T-cells and also an increased proportion of CD4/CD8 double-negative T-cells.

**Figure 2:**
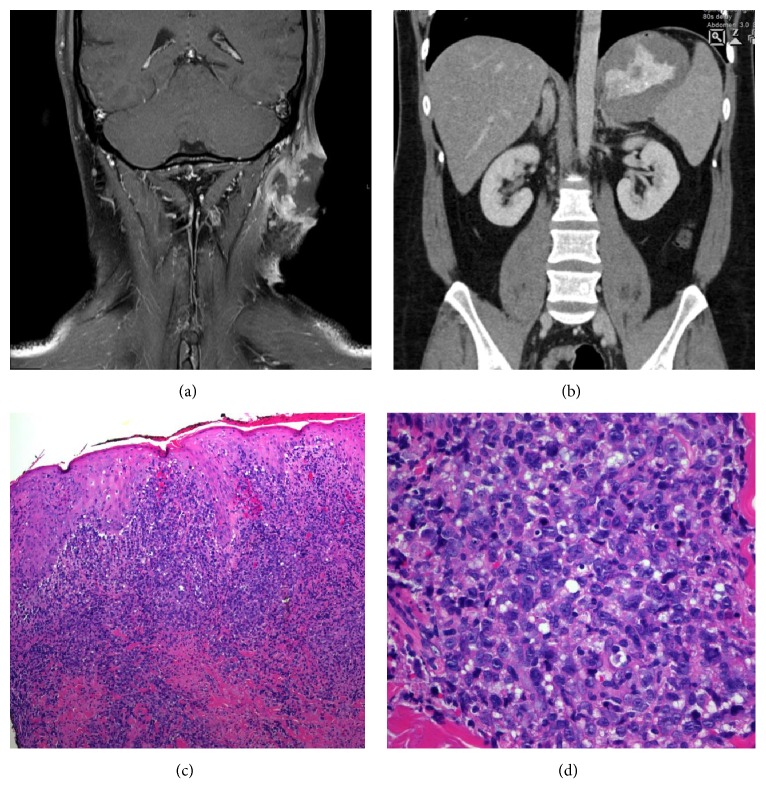
Affection of facial muscles and internal organs. (a) MR of the neck showing a large ulcerating tumor on the left side with infiltration into nearby muscles. (b) CT of the abdomen showing massive thickening of the ventricular wall due to lymphoma with infiltration also into the spleen and left adrenal gland. (c, d) Skin biopsy from the neck lesion showing sheets of lymphoid cells throughout dermis, with large pale gray nuclei with prominent nucleoli (H&E, 100x and 400x, resp.). On immunohistochemistry, the cells were positive for CD20, Pax5, Bcl-2, MUM1, and EBERISH and negative for CD3, CD4, CD5, CD8, CD30, CD10, Bcl-6, and cyclin D1. Ki-67 showed a proliferative fraction of 70–80% of tumor cells. The lesion was classified as diffuse large B-cell lymphoma.

The patient had skin lesions from 2 years of age, affecting his face and extremities and to a lesser degree his truncus. The lesions appeared as purple plaques of the skin with variable thickness evolving into deep atrophic scars over time. There had been numerous biopsies over the years showing an uncharacteristic massive lymphoid infiltration dominated by CD8+ lymphocytes and histiocytes and with some granulomas (Figure 1). In lack of a more precise histopathological diagnosis and considering the clinical presentation, a diagnosis of sarcoidosis was given. Despite aggressive immunosuppressive therapy including corticosteroids, methotrexate, azathioprine, and anti-TNF agents, the lesions gradually evolved although in a slow and undulating manner. In a reevaluation of the skin biopsies, the pathologists found a diffuse lymphohistiocytic infiltrate dominated by CD8^+^ T-cells with several different monoclonalities as well as epithelioid cell granulomas. They concluded with a T-cell dominated lymphoproliferative state associated with a form of primary immunodeficiency, for example, a* RAG*-mutation. Meanwhile the patient underwent carmustine therapy on the suspicion of mycosis fungoides without any apparent clinical effect. One year before presentation at Oslo University Hospital, the patient was found to have a diffuse large cell B-cell lymphoma (non-GC-B type, EBV+) in a tumor of the neck and at the same time another different B-cell lymphoma (unclassifiable, with features intermediate between diffuse large-B cell lymphoma (DLBCL) and classical Hodgkin lymphoma) preauricularly (Figure 2). PET-CT showed a possible lymphoma in the right lung, and as such he was staged as IVA, aaIPI 1. He underwent chemotherapy (R-CHOEP 14x 6 + 2R) [10, 11] and radiation therapy against residual disease and went in complete remission with a negative PET-CT. His skin lesions, however, got worse throughout the chemotherapy and radiation therapy and continued to evolve with infiltration into the masseter muscle in the face and a similar histopathological picture was found in lesions diffusely infiltrating the liver and possibly spleen. The patient had no history of recurrent infections, autoimmunity, or other manifestations of a primary immunodeficiency beside his skin lesions and lymphoma.

At presentation at Oslo University Hospital, he was immunologically characterized by a slight hypogammaglobulinemia (IgG 6,1 g/L) and absence of B-cells in peripheral blood considered to be secondary to rituximab treatment (Table 1). However, low numbers but not absence of B-cells in previous bone marrow biopsies had been noted. He did not receive any immunoglobulin therapy. He was slightly lymphopenic (0,8 × 10^9^/L) and had low levels of CD4+ cells (177 × 10^6^/L) with an inversed CD4/CD8 ratio of 0,63. There was an absence of naïve CD4+ T-cells and recent thymic emigrants. His CD8+ cell pool was expanded but had a normal phenotype. He had normal levels of double-negative T-cells and gamma-delta T-cells. Overall the T-cells seemed to be strongly activated with half being HLA-DR+. We have no data on proliferative response to mitogens or specific antigens.

There was a strong clinical suspicion of an underlying immunodeficiency but DNA-sequencing covering exons and introns to the* RAG1/RAG2* gene did not reveal any mutations. Samples were taken for whole-exome sequencing, but while waiting for the results, the patient had a DLCBL-like relapse in the gastroventricular mucosa engaging pancreas and spleen with infiltration of irregularly shaped histiocytic cells and T-cells monoclonal for the TCR-gamma chain. Dose-reduced chemotherapy was initiated (isophosphamide, methotrexate, and etoposide), but after 3 days of therapy, a near catastrophic gastrointestinal bleeding occurred from the tumor. He went through extensive surgery and we received report of a homozygous missense mutation in exon 8 of the Artemis-coding gene* DCLRE1C* (NM_001033855.2: c.632G>T, p.G211V), while he was still in the intensive care unit. Sanger sequencing confirmed homozygosity in patient DNA and heterozygosity in his father and one of his siblings, while his mother was not tested. Fibroblastic culture furthermore revealed radiosensitivity consistent with an Artemis defect. The genetic variant has previously been reported as disease-causing in a patient from a patient cohort with radiosensitive SCID and Omenn syndrome [12]. It has not been observed in databases for normal variation (ExAC and gnomAD) [13].

The affected amino acid is located in the *β*-CASP domain of DCLRE1C [14]. Predicted secondary structure suggests that the amino acid is located in a six-amino-acid-sized stretch between a helix and a strand. The amino acid is highly conserved both between species and in members of the *β*-CASP domain.

The patient was again evaluated by PET-CT and considered in complete remission after surgery and referred for HSCT. He had no severe comorbidity, and the now increasingly aggressive nature of his immunodeficiency made the case for HSCT well founded. None of his siblings were HLA identical and no matched unrelated donor could be identified. For practical reasons, availability as well as identical ABO type, we decided to choose his haploidentical father as the preferred haploidentical stem cell donor. The parents' heterozygosity for the* Artemis *mutation was considered irrelevant due to the recessive nature of the trait and their apparently normal immune function.* Artemis* mutated immunodeficiencies in general are likely to tolerate myeloablative conditioning, which gives the best long-term immune reconstitution with thymopoiesis and B-cell function. The options of (A) conditioning with alemtuzumab, treosulfan, fludarabine, and thiotepa using a TCR-alpha and -beta depleted graft versus (B) using a T-cell replete marrow graft after cyclophosphamide 14, 5 mg/kg days −6 and −5, flu 30 mg/m^2^ days −6 to −2 (5 days), and 200 cGy TBI day −1 [15] with the addition of busulfan 3.2 mg/kg/day, days −4 and −3 to achieve myeloablation [16] as conditioning and posttransplant cyclophosphamide 50 mg/kg days +3 and +4 and tacrolimus and mycophenolate mofetil as graft-versus-host-disease (GVHD) prophylaxis [15] were discussed. We chose the latter option for our patient, and he received BMSC, only 1,7 × 10^8^ NC/Kg, and stable engraftment was achieved at day +18. Donor chimerism was >99% at day 100. The posttransplant course was uncomplicated except for two episodes of CMV reactivation that was preemptively treated.

On Day 150, however, the patient was referred to hospital with fever and lung symptoms with CT scans demonstrating extensive lung infiltrates. Bronchoscopy with bronchoalveolar lavage was performed showing a positive PCR reaction for* Pneumocystis jirovecii*, and while the immunofluorescence test was negative, a* Pneumocystis jirovecii* pneumonia (PCP) was highly suspected. PCP prophylaxis with trimethoprim-sulfamethoxazole had been prescribed but the patient had for unknown reasons only taken this on and off. Further tests showed a positive Herpes simplex virus PCR reaction in plasma, and* Staphylococcus aureus *and* Candida albicans* were found in blood cultures. The patient was extensively treated with antibiotics including high-dose trimethoprim-sulfamethoxazole as well as corticosteroids and acyclovir. Sadly, however, he did not respond to this treatment and died on day 167. Autopsy was unfortunately not done.

## 3. Discussion

This case of a hypomorphic* Artemis* mutation highlights the progressive nature of this disease, gradually presenting new complications as the clinicians strive to keep clinical control. Mutations in the* Artemis/DCLRE1C* gene have traditionally been associated with SCID and Omenn syndrome, but recently various mutations have been shown to be associated also with milder forms of immunodeficiencies, including hypogammaglobulinemia [3, 4]. While dysfunction of the* Artemis* gene is less destructive to the recombination of T-cell and B-cell receptors than RAG deficiencies, the ubiquitous distribution of the protein in all cells of the body makes the clinical consequences both wider and less predictable [2]. The mutation found in our patient has previously been reported as causing SCID-like disease but the clinical and immunological characteristics of these patients are not known [12]. There is a known association between Artemis deficiency and lymphomas, as demonstrated by early reports of aggressive B-cell lymphomas in patients with hypomorphic* Artemis* mutations, as well as mutations in DNA-repair genes in B-cell lymphomas [8, 17, 18].

Leaky SCIDs and other forms of combined immunodeficiencies with an indolent but severe prognosis represent a special challenge to geneticists, immunologists, and clinicians involved in the care of these patients. In our case, the patient had more than 20 years of slowly progressing skin lesions while being generally in good health and with no apparent immunodeficiency.

The patient had unique skin manifestations and even if sarcoidosis of the skin can present itself with a wide range of lesion from papules to plaques and scarring, it is unusual in small children. The histopathological pattern with lymphohistiocytic skin infiltrates with or without granulomas is unspecific but is reported in several different primary immunodeficiencies, including deficiencies of Artemis [19], and can be an early finding. This patient later developed overt B-cell lymphomas of different subtypes. They were all EBV-positive, similar to what is often seen in immunodeficient patients in the posttransplant situation. Both histopathological settings could therefore alert the observant pathologist in cooperation with clinicians in the direction of immunodeficiency as an underlying cause. At the time an immunodeficiency was suspected he had already been through massive medical treatment and an assessment of his “naïve” immunological state is hard to make. The patient had a mild hypogammaglobulinemia that could have been secondary to both his previous treatment and his immunodeficiency. B-cells can be absent from peripheral blood for several years after treatment with rituximab but are not always accompanied by significant hypogammaglobulinemia.

The diagnosis of SCID is transforming as more and more centers introduce screening methods that will detect major T-cell defects even before clinical symptoms become apparent, as is possible in this case.

The introduction of whole-exome sequencing to clinical immunology has transformed not only the diagnostics but also the treatment of primary immunodeficiencies. In our case, the strong suspicion of a recombination defect was confirmed and made the decision to proceed with a transplant easier, even without a matching donor. Furthermore, even if the clinical basis for a transplant could be considered clear without the exact diagnosis, the finding of a recombination defect and the subsequent radiosensitivity assay had a major influence on the choice of protocol and conditioning needed. Time was of the essence, and the value of a prompt diagnosis in this setting cannot be overestimated.

Data from our center indicate that whole-exome sequencing leads to revision of the clinical diagnosis in more than half of the patients and management was directly altered in nearly a quarter of families based on molecular findings [20]. The major obstacles for the wide implementation of high-throughput sequencing in diagnosis of primary immunodeficiencies are the high price and limited capacity for sequencing and interpretation. Both continue to improve, but the immense value of this diagnostic tool in the care of these highly complex patients should make this happen sooner rather than later.

## Figures and Tables

**Table 1:** Hematologic and immunologic characteristics.

	Before transplant^*∗*^	Day 30	Day 150
Blood counts			
Hemoglobin (g/dL)	12,1	7,9	10,0
Thrombocytes (×10^9^/L)	240	39	82
Leukocytes (×10^9^/L)	3,9	3,2	14,1
Lymphocytes (×10^9^/L)	0,8	0,5	6,7
CD4 (×10^6^/L)	177		
CD8 (×10^6^/L)	277		
CD19 (×10^6^/L)	<5		
Immunoglobulins			
IgG (g/L)	6,1		2,8
IgM (g/L)	0,1		<0,1
IgA (g/L)	0,4		<0,1
Chimerism			99%^*∗∗*^

^*∗*^Pretransplant data from first presentation at Oslo University Hospital. ^*∗∗*^Chimerism data at day 100.

## Data Availability

All available data is included in the manuscript. The full transcript of the genomic analyses cannot be published due to legislative regulations.
